# ‘You've come to children that are in care and given us the opportunity to get our voices heard': The journey of looked after children and researchers in developing a Patient and Public Involvement group

**DOI:** 10.1111/hex.12904

**Published:** 2019-05-21

**Authors:** Hayley Alderson, Rebecca Brown, Debbie Smart, Raghu Lingam, Gail Dovey‐Pearce

**Affiliations:** ^1^ Institute of Health and Society Newcastle University Newcastle Upton Tyne UK; ^2^ Faculty of Medicine University of New South Wales Sydney New South Wales Australia; ^3^ Northumbria Healthcare NHS Foundation Trust North Shields UK

**Keywords:** looked after children, Patient and Public Involvement, qualitative research

## Abstract

**Background:**

Looked after children and care leavers (denoted as LAC) are often described as a ‘hard to reach' group of young people, and their voices are rarely sought to inform academic research.

**Methods:**

This paper reports on experiences and reflections of a group of children and young people and academic researchers who developed a Patient and Public Involvement (PPI) group that was set up in the context of an ongoing health service intervention trial with LAC.

**Setting and participants:**

Eighteen qualitative semi‐structured interviews were conducted with seven LAC, the participation officer within a North East Children in Care Council and the four researchers involved in developing and facilitating the PPI group. PPI sessions (n = 9) each approximately 1 hour in length were conducted over an 18‐month period.

**Analysis:**

The qualitative interviews were transcribed verbatim. Thematic analysis was used to analyse the data, and direct quotes are used within the paper.

**Main outcomes:**

The LAC used the PPI group to produce a 5‐minute video to highlight why they think young people should be involved in research. Overall findings suggested that it was feasible to develop a research‐related PPI group with LAC. Findings from the research were used to co‐develop ‘top tips' of working with vulnerable young people such as looked after children.

**Conclusion:**

This paper has shown that PPI with LAC can be done if a co‐production approach to research is taken. It also suggests that assumptions regarding the capabilities of young people as researchers need to be re‐evaluated.

## 1 BACKGROUND

Looked after children in the UK are young people aged 0‐18 years who have been placed under the legal care of the state, largely due to a history of abuse and/or neglect. They are termed as living in Out of Home care in Australia and the United States. There are almost 73 000 children in the UK care system.^1^ This represents 62 children per 10 000 of those aged under 18.^1^ Care leavers are young adults who are no longer looked after, but are still entitled to support from their local authority. Care leavers can be of age 16‐25 depending on their situation, such as whether they are in education, but are typically aged 18‐21.^1^

Looked after children and care leavers (henceforth LAC) are often disadvantaged, and they are more likely than their peers to have experienced Adverse Childhood Experiences.^2^, ^3^ LAC, aged 11‐19 years, have a fourfold increased risk of drug and alcohol use than children not in care,^4^ and 50% of those in care meet the diagnostic criteria for a psychiatric disorder, compared to 10% of non‐care children who have mental health issues.^5^ At age 16, the average attainment score for LAC is 22.8, compared to a score nearly double that for children not in care.^6^, ^7^, ^8^, ^9^

## 2 RATIONALE FOR CARRYING OUT PATIENT AND PUBLIC INVOLVEMENT IN RESEARCH

The National Institute of Health Research (NIHR) defines involvement as ‘research being carried out “with” or “by” members of the public rather than “to,” “about” or “for” them'.^10^ Patient and Public Involvement (PPI) should actively involve members of the public in research, planning what to research and how it should be done.^11^ ^, p.5^ Ideally, a co‐production approach should be taken, in which researchers, the public and professionals work together to share power and responsibility for the whole research project.^12^ Recent NIHR guidance identifies five key principles for ‘co‐production of research projects'.^12^ The principles are sharing of power, including all perspectives, respecting and valuing everyone's knowledge, reciprocity and building and maintaining relationships. Research shows that PPI identifies different perspectives regarding research topics^13^ and allows more ideas to be considered than if consultation was aimed at a small group of like‐minded people.^14^, ^15^, ^16^ Alongside recognizing that PPI improves research, people have a right to be involved in things that affect them, and this is formalized by the United Nations Convention on the Rights of the Child (Article 12) that states all children have the right to be involved in decisions that affect them.^17^

In research, PPI work has a tendency to focus on specific health conditions, and experiences of attending health services.^18^ PPI groups tend to be (made up of people who are) white, middle class and retired.^19^ ^, p. 21^ Much PPI is conducted with adults and often underrepresents, daytime workers, people from lower socio‐economic backgrounds without a university education and ethnic minorities.^20^, ^21^ Additionally, PPI work often ignores the views of children and young people (CYP), and their voices are largely absent within the design of academic research contrary to the United Nations Convention on the Rights of the Child.^17^

Guidelines are available to inform the involvement of CYP in PPI groups and research.^11^, ^22^, ^23^ The most popular model for children and young people's involvement in health research is the Young Person's research Advisory Groups, for example the Generation R Alliance.^24^ Whilst there is recognition of the importance of obtaining multiple perspectives, the inclusion of seldom heard, marginalized and socially excluded groups continues to pose challenges.^25^, ^26^, ^27^

This paper aims to make a contribution to filling the gap regarding involving marginalized populations of CYP in research. Dominelli (2005) states that ‘even the most premeditated forms of empirical qualitative research tend to be unpredictable and somewhat “messy”',^28^, ^29^ ^, p. 229^ the prospect of engaging a population whom is transient and surrounded by multiple complexities can be daunting. This coupled with researchers having limited time and finite resources to dedicate to engaging this group of young people results in the voices of LAC being underutilized in the research process.^29^ However, despite being challenging, it is necessary to involve LAC at all stages of the research process to ensure that they have an opportunity to be involved in decisions that affect them^17^ and to strengthen the research process, making outcomes more credible and relevant to LAC individuals and wider policy decisions.^24^

## 3 METHODS

Semi‐structured interviews were conducted at two separate time points (prior to commencing sessions and within the final session) with LAC. This method was chosen for its strength in exploring participants' experiences, feelings and perspectives. A semi‐structured approach used a topic guide, but allowed the researchers to remain flexible enough to explore unforeseen areas of discussion. The first semi‐structured interviews took place with seven LAC. Interviews explored LAC's understanding of the term ‘research', how they felt they could contribute to a research project and their expectations and feelings about working with researchers. Interviews were completed by the researchers facilitating the PPI project. LAC volunteered to take part in the interviews conducted within a Children in Care Council (CICC) session; the interview took place in a separate room to ensure confidentiality.

The researchers involved in the PPI project were interviewed (n = 3) twice by an independent researcher. The first interviews aimed to capture the researcher's previous experience of being involved in PPI work and their hopes and expectations of the project prior to it commencing.

Upon completion of the PPI work, a second qualitative interview took place with participants (LAC [n = 4], the CICC's participation officer [n = 1] and researchers involved in the project [n = 3], two of the researchers were the same as those at initial interview, whilst one researcher had changed). The final interview with LAC included participants who were present within the CICC session and had been involved in the PPI work. Four of the original seven involved in the first interview took part (two LAC had relocated outside of the study area, and one young person was not attending the CICC meetings at this point due to health issues). Interviews discussed participants' experience of being involved in the research, if their expectations had been met and whether anything needed to change to facilitate involvement in future research projects. The interviews also aimed to increase our understanding of the practicalities and process of working with LAC.

Prior to each interview, written informed assent/consent was obtained from all researchers, the CICC participation officer and LAC, inclusive of consent from the corporate guardian for LAC under 16 years of age. Interviews were carried out by experienced researchers, audio‐recorded and transcribed verbatim. Transcripts were anonymized, and a participant key was stored separately.

Looked after children and care leavers were given a £10 voucher for each session they engaged with to demonstrate that their contributions are valued and their expertise respected.^30^

Study data have been analysed using thematic analysis.^31^ The constant comparison method was used,^32^ an iterative process comparing data within and across groups, highlighting similarities and differences. Direct quotes included came from LAC, researchers and the CICC participation officer. Pseudonyms are used throughout to protect participants' identities.

The findings from this project explored pre‐conceptions about the ability to engage and work with this group of young people.^33^, ^34^ When reporting the study, the team ensured that the core items identified in the Guidance for Reporting Involvement of Patients and the Public (GRIPP) short form were adhered to.^35^

### 3.1 Setting up and running the LAC PPI group

This PPI project was set up in the context of an ongoing health service intervention trial called Supporting Looked After Children and Care Leavers In Decreasing Drugs, and alcohol (SOLID). Within the initial set up phase of the SOLID trial, the research team consulted with LAC and received feedback that they would like to have been involved at an earlier stage to more fully influence the research agenda. This drove forward a satellite piece of work, to understand more about how to work closely with LAC to develop an ongoing research programme. We approached an already established CICC to determine whether LAC would like to be involved in a PPI project. In the UK, each local authority has a CICC specifically designed to give children in care and care leavers an opportunity to have a voice and give their opinions on how the council should run its Children's Services. The CICC participation officer acted as a mediator and arranged for researchers to attend a CICC meeting. LAC were asked to register their interest in taking part in the PPI project with the CICC participation officer. Once confirmation of interest was received, a series of sessions were set up in collaboration with all LAC.

The CICC participation officers' role was to organize mutually convenient times for LAC and researchers to meet. Sessions were organized to accommodate the requests of the LAC regarding times, duration and venue. LAC requested that researchers attended the already established CICC meetings on dates convenient to them; that is, PPI sessions did not conflict with their other commitments. During sessions, a separate room was provided, so that individuals could either take part in the PPI work or stay within the CICC meeting, exercising their right to be involved in the PPI work and equally their right not to be involved.^27^, ^36^ They were welcome to enter and leave sessions whenever they wanted. Throughout the project, 11 LAC participated and nine sessions were held over an 18‐month period. Attendance varied between two and seven LAC with two of the 11 LAC attending every session.

The LAC were 15‐19 years old, all were white, in keeping with the majority demographic of the local area, and resided in North East England. The LAC (six male and five female) resided in foster placements, residential children's homes and independent living.

The project enabled LAC to establish their own project using methods of their own choice. It was established through informal discussions and the initial semi‐structured interview that LAC would like to produce a video. This video could be used to inform other CYP and academics what research is and why they think young people should be involved in research projects. LAC took part in sessions according to their own interests and abilities.

Sessions were in two parts: the first involved a facilitated exploratory discussion to consider components of ‘academic research', within which different types of research, that is, qualitative and quantitative methods and types of data collection such as interview/focus groups and surveys were discussed. The second part of the session was used to video record the group taking part in activities and practising skills such as interview techniques and mock focus groups that could be used within the final video production.

The last session was used to showcase the final video, produce the top tips and provide certificates of attendance to all LAC.

### 3.2 Findings and lessons learnt

The key contribution and shared learning from the PPI was in the expertise brought by the research team and the lived experience of the LAC involved. A number of themes emerged, and they have provided learning regarding the logistics and the processes of involving LAC in academic research.

When discussing their motivation for being involved, there was a desire to learn new skills.

Learn something new innit? Obviously I've never really done that kind of stuff before

(Paul, LAC)

Being involved in new things provided an opportunity to receive a certificate to record their achievement, and there was a definite focus on the future.

Having a certificate, you've always got it so you could always be reminded of the good things you've done. Stuff like that's really good to have on CV's.

(Lisa, LAC)

### 3.3 Involvement as a fluid and evolving process

The project was fluid, and the idea for the project was led by the LAC and evolved from discussions;

You could do a mini documentary on like how…..yous could have like a bit that explains why yous are doing the research. Like you could include some of the young people's point in it, it would be really good.

(Lisa, LAC)

Due to the voluntary nature of the group, researchers had to be mindful of creating a relaxed environment whilst also progressing towards creating an end product;

Our group are the less engaged and harder to engage, so you've got to make it really flexible for them. People need to remember that actually they're volunteering their time, so they don't have to be there.

(Joanne, Participation Officer)

Each session required active facilitation with researchers balancing a goal‐focused approach alongside being responsive to the young people;

Whoever wanted to be involved in that session, and take a specific role, they were encouraged to do so– and did.

(Rachel, Researcher)

There was a strong and repeated request for sessions to be interactive. Researchers needed to be pragmatic when thinking about the structure and content of sessions and what was realistically achievable. A variety of skills were adopted to keep participants engaged;

Do like, activities basically, because sitting round a desk and talking isn't very engaging.

(Sarah, LAC)

### 3.4 Building and maintaining relationships

The ability to engage LAC and establish a working relationship was key to the success of the project, and it had to be done face to face to truly engage participants;

Once they've met you a couple of times, they start to engage a bit more. Building a relationship with them is really, really important.

(Joanne, Participation Officer)

Having the opportunity to meet with LAC over an 18‐month period enabled LAC to relax and build up a rapport with researchers;

The group in general, it wasn't too formal, it was just a place where you could talk

(Sarah, LAC)

### 3.5 Awareness of power

Generally, LAC are perceived by society as vulnerable, hard to engage and in need of protection,^9^ and therefore, they miss the opportunity to be involved;

Some people will treat us differently but you have come to us to ask us whether we want to do it. Rather than just going to a group of young people, “Right, do you want to do this?” you've come to children that are in care and given us the opportunity to get our voices heard

(David, LAC)

Once LAC were familiar with the researchers, they articulated themselves clearly and CICC members did not stereotypically present in a ‘vulnerable' way;

Sometimes we have these misconceptions of looked after young people. Social workers think they are so vulnerable and they need protecting from this and that and sometimes they haven't got the right skills to communicate with professional and adult people in a way we would want them to but as a researcher I am seeing something totally different.

(Mel, Researcher)

Mel witnessed LAC volunteering to be involved in the study, and they were assertive during activities and confident in articulating themselves when talking to the researchers.

In direct response to LAC's ability to express themselves, researchers had a heightened awareness of maximizing opportunities for LAC to shape the project. This was reinforced when the participation officer stated that LAC wanted to feel;

“Actually I've got a bit of a voice and I've got a bit of power,” a lot of these young people have been through being powerless, really, and had a lot of things done to them.

(Joanne, Participation Officer)

Researchers wanted to support LAC to be actively engaged in the project, working towards a common goal. Despite trying to engage LAC on an ‘equal' level, by encouraging discussions led by the LAC and handing control to the group regarding which skills to practice and which elements to video record, some participants still conformed to a more traditional teacher‐student role;

I think there was still the age of the power dynamic. I was sitting in front of a 14‐year‐old girl who was at school who saw me as asking her questions to get the right answer from her and it wasn't like that.

(Lyndsay, Researcher)

### 3.6 Respecting everyone's knowledge and skills

Researchers had to be vigilant of external factors affecting LAC and how they impacted upon individuals and affected group dynamics. The decision to make the PPI group ‘open access' rather than a closed group enabled LAC to attend when possible, without feeling excluded if they missed sessions;

“Actually, they've got so much going on that they might have all the will in the world to be involved in this project, but life just takes place.” By keeping turning up, it gave them another opportunity to come back when they were ready.

(Grace, Researcher)

The research team ensured that there was not any pressure to undertake roles they were not comfortable with and LAC appreciated this level of choice and control over their input;

I don't want to be on camera so you gave me the opportunity of videoing it instead….so you gave all of us a choice of whether we want to be on camera or not

(David, LAC)

### 3.7 Reciprocity in the PPI project

Looked after children and care leavers received a certificate to record their achievement and acknowledged that the skills they had acquired were transferable;

I mean interviewing skills, like life skills, you know, I can take away from that and just the different formats of research that you can do

(Sarah, LAC)

Researchers working on the project were all white British females, in their mid‐30s. All researchers had previous experience of working with young people who accessed drug and alcohol services and young carers support. Despite previous experience, researchers continued to develop their skills;

It's quite humbling, that… They can bring you to your knees, in a way, by off‐hand comments or not doing what you thought was going to happen. Having to learn, I guess, to improvise.

(Lyndsay, Researcher)

This study reinforced that it is essential for researchers working with this participant group to develop skills such as resilience, patience and tolerance. A commitment to understanding the factors affecting the lives of LAC at the same time as working sensitively and with tact is imperative.

At the end of the project, LAC wanted to understand how their input had influenced the research project;

It's all very well and good doing a project, but then if you don't know how it turns out, you know…….was it totally useless, sort of thing?

(Amy, LAC)

Looked after children and care leavers and the participation officer voiced a frustration when external people parachute in and conduct tokenistic consultations with members of the CICC without providing feedback as to how their input had influenced anything.

To ensure this did not happen in this project, LAC helped to edit the final video. Once completed, they watched the video and approved use at dissemination events. LAC felt the video they developed could be used in multiple settings to raise awareness of involving CYP in research. This was a positive output for LAC to have been involved in.

### 3.8 Producing ‘top tips' of working with young people

The group made recommendations of the ‘top tips' for working with LAC and other marginalized CYP in research. The top tips were devised by taking part in a group exercise where LAC individually wrote down tips they thought were important, and they then worked together to co‐produce the ten most important items for consideration. The top tips were divided into suggestions around organizing and running a session.

#### 3.8.1 Organizing a session

Many of the tips are relevant and important to consider for any PPI group but are even more so for children with care experiences.

1. **Provide transport to sessions**—Although transport can be a barrier to any involvement, for LAC and care leavers especially, access to transport can be extremely problematic. Many care leavers live independently with limited finances. They do not have the finances to pay for public transport in order for it to be reimbursed. Therefore, it was pivotal that transport was provided for this particular group of young people or an advanced travel pass issued.
2. **Interactive sessions—**For the majority of the LAC involved in this project, participating in groups or research implies that it is in addition to school, college or employment. They did not want sessions to feel like an extension of their education by having a ‘teacher‐student' feel to the sessions. Additionally, it is known that many LAC have a reduced level of literacy and behavioural diagnoses of conditions such as attention‐deficit hyperactivity disorder.^37^, ^38^ It was important that sessions were interactive and shaped around individual's needs.
3. **Keep sessions short**—Sessions need to be a maximum of 1 hour, including comfort and refreshment breaks. Researchers need to gauge each session on its own merit and be prepared to make adjustments if LAC are starting to disengage to reduce the likelihood of young people thinking negatively about their experience of involvement.
4. **Meetings after school**—Timing of sessions was important, and LAC suggested 4‐4:30 pm as an optimum time to start sessions. This allows LAC time to travel to sessions after completion of their daily commitments but would not interfere too significantly with ‘tea time' or other responsibilities they have.
5. **Location needs to be familiar**—The location was important for LAC whom explained that they are often exposed to numerous different workers and appointments in different locations. This can be anxiety provoking. Therefore, if possible, involvement in PPI/research should take place in a familiar location. This was reinforced by LAC stating that involvement in research could be daunting; if the location was already familiar, it made participating easier.

#### 3.8.2 Running a session

1. **A familiar face**—A well‐known face helps LAC to overcome potential insecure attachments^27^ that they may have experienced/may be experiencing by nature of being in care. The participation officer organized the room bookings and also helped to maintain contact with LAC, sending reminder text messages, acting as a sounding board if they had any questions and being available within PPI sessions to provide a familiar face and being a source of positive support throughout the project.
2. **A researcher who understands**—LAC within the group stated that they wanted a researcher that had an understanding of their circumstances and an awareness of the care system and the complexities that they faced. Researchers had to be able to engage with LAC on their terms, be non‐judgemental and sensitive in their approach.
3. **Teach us a new skill**—PPI should be designed so that participants learn a new skill, as Dunn (2018) also highlights when working with a group of young people experiencing depression.^39^
4. **Provide a certificate**—A certificate of attendance was important for this group, especially for some LAC who due to disrupted education have limited formal qualifications.
5. **Incentives**—Incentives are always welcome as a sign of appreciation.^30^ What was interesting for this group of LAC was that the incentive was only useful if it was relevant to them. For all LAC, but care leavers especially incentives of food vouchers were most relevant to them. This would enable them to purchase food, which sometimes they struggle to afford.

## 4 DISCUSSION

The current paper gives an example of how to develop and engage a group of LAC in a time‐limited PPI project. The findings and top tips reflect the practicalities of working with an under‐represented group, whom often present with a range of competing demands and needs. This project has highlighted that involving LAC in academic research can result in concrete outcomes and has key impacts. It has also highlighted that the involvement process needs to be understood and carefully facilitated, for the desired outputs and impacts to be realized. In line with Dovey‐Pearce's paper, this project suggests that assumptions regarding the capabilities of CYP as researchers need to be re‐evaluated.^40^

This paper highlights that involving LAC in a PPI group to inform research has considerable potential to be mutually beneficial; however, there is little evidence available regarding successful examples of the *process* of their involvement or its *impact*.^41^, ^42^ Brett et al's^43^ systematic review provided the first international evidence of PPI impact that had emerged at all key stages of the research process. However, their review concluded that much of the evidence base concerning impact remains weak and needs significant enhancement. This is certainly true for LAC who are recognized as a marginalized and socially excluded group whom are less likely to be involved in research. It is also recognized that when links are successfully established with individuals such as LAC, they tend to feel over‐consulted, so a careful balance has to be achieved.^44^, ^45^

With that in mind, this paper highlights important factors to be considered when undertaking PPI with LAC or groups of under‐represented young people. Unsurprisingly, many of the findings are relevant and important to consider for any PPI/research group with young people and the suggested findings are closely aligned with the guidance produced by INVOLVE,^22^ National Children's Bureau,^46^ the NIHR,^11^ the NHS^23^ and other authors regarding CYP's involvement in research or PPI groups.^45^, ^47^ However, when comparing guidance documents, there are some significant differences regarding the priorities of LAC in their ‘top tips' for involvement. What deviates from the more generic guidance is that the LAC involved in this project placed transport, logistics of the sessions and location at the top of their checklist. On reflection, the ‘top tips' were devised at the end of the project, and therefore, the usual ideas of respect, involving LAC from the beginning, providing training and giving feedback may have featured more heavily had we not already successfully managed to do that within the project.

The idea of involvement in research being mutually beneficial and showing respect to CYP is present in the generic PPI guidance, as is the requirement to provide training and support through the research^48^ and feedback once the project has concluded. However, the reasons behind some of the requirements are different and it is important to acknowledge the subtleties and understand the specifics of *why* some ideas are important when considering a LAC population.

Young people can often be viewed as being relatively powerless due to their levels of capacity and competency due to their age.^33^, ^49^ The term ‘power' can have negative connotations when considering more marginalized groups due to negative forms of power, such as domination. In terms of power in relation to participatory research, it is argued that this should not lead researchers to avoid discussions about power or strive for absolute equality but understand that power differentials exist and enablement and emancipation can allow people to choose to enact their inherent powers and capabilities.^50^ Communication and negotiation are key in recognizing power differentials and discussing how and when decisions can be taken and by whom.^51^ Within this project, LAC asserted their power by either electing not to attend sessions, attending but deciding not to participate in the research study and self‐selecting what to share within the sessions. A conscious effort was made for LAC to be mutually involved in the research process. For researchers, this shift in the power dynamic was essential for an effective group and was encouraged. The researchers had to consider possible ways of redistributing power, such as LAC deciding what the project should look like and the format it should take (producing a video) to maximize involvement opportunities and align as closely as possible to the co‐production principles discussed earlier. Researchers believe that PPI in this project could be classed as ‘co‐production', the main challenge was sharing power and we do not think that absolute equality was achieved as throughout the project researchers introduced ideas and prepared materials to facilitate discussions.

There is a need to understand that tokenism may be experienced by LAC as oppressive as for many LAC the receipt of statutory care is involuntary.^52^ The study reinforced the importance LAC place on feeling respected and that their voices are heard because they have often lacked control in other areas of their lives.^52^, ^53^, ^54^ The attempt to genuinely give LAC a voice translated within this project to a co‐produced video and top tips wherein LAC developed the idea of making a short film within the CICC meetings. The final film indicated to other potential audiences that the research team had successfully managed to give LAC a voice.

Throughout the project, participatory activities improved and LAC's confidence appeared to improve with individuals taking on roles they had previously declined to and asking to be involved in future projects. This is in line with previous findings within which participants involved in PPI have reported, feeling listened to^43^, ^55^, ^56^, ^57^ and feeling valued.^58^ Additionally, being involved in the project helped to provide an experience to add to their CV, making them more desirable when they sought employment.^57^ A certificate of attendance was important for this group, especially for some LAC, who due to disrupted education have limited formal qualifications. The project provided an opportunity for researchers to follow the key co‐production principles and show that LAC can successfully take part in extracurricular activities.

## 5 STRENGTHS AND LIMITATIONS

The opportunity of engaging with an existing group where LAC are represented and the availability of a familiar face (participation officer) helped to overcome some of the barriers to engagement and was undoubtedly paramount to the success of this research project.

A limitation of the study is that the number of LAC involved in the research was small and all LAC resided in the same geographical location. The project was extremely resource intensive for a small number of LAC; this limits the potential for generalization and may make the study difficult to replicate.^59^

## 6 CONCLUSION

This paper has described the process of involving LAC in developing and engaging in PPI in academic research. This study has shown that PPI with this group of young people can be done, if researchers have enough time, resources and willingness to work at the pace of the participants. Further work is needed which ensures that LAC have an opportunity to co‐produce research ideas and work on projects and develop an ongoing research strategy for other LAC. Future work needs to accurately assess the impact of PPI work undertaken.

## CONFLICT OF INTERESTS

The authors declare that they have no conflict of interests.

## AUTHOR CONTRIBUTIONS

All authors have made an intellectual contribution to this research paper. HA, RL and GDP conceptualized the initial idea of the study. HA wrote the first draft of the paper, and all authors (RB, DS, RL and GDP) have input into drafts and have read and approved the final version of the manuscript.

## ETHICAL APPROVAL AND CONSENT TO PARTICIPATE

This study was undertaken as part of the ongoing PPI element of the SOLID trial, and therefore, the same ethical approval was applied. The SOLID study was granted a favourable ethical opinion by Newcastle and North Tyneside 1 NRES Committee (16/NE/0123). Newcastle University acted as trial sponsor. The SOLID Management Group have been responsible for ensuring the appropriate and timely implementation of the study.

Written informed consent was obtained for all participants (LAC, researchers and CICC participation officer), inclusive of assent from LAC under 16 years of age and consent from their corporate guardian.

## AVAILABILITY OF DATA AND MATERIAL

The datasets used and/or analysed during the current study are available from the corresponding author on reasonable request.
